# RIG-I and cGAS mediate antimicrobial and inflammatory responses of primary osteoblasts and osteoclasts to *Staphylococcus aureus*

**DOI:** 10.1128/mbio.03971-24

**Published:** 2025-03-26

**Authors:** Erin L. Mills, Samantha R. Suptela, Mary-Kate Key, Ian Marriott, M. Brittany Johnson

**Affiliations:** 1 Department of Biological Sciences, University of North Carolina at Charlotte, Charlotte, North Carolina, USA; 2 Graduate Division of Biological and Biomedical Sciences, Emory University, Atlanta, Georgia, USA; St. Jude Children's Research Hospital, Memphis, Tennessee, USA; University of Illinois Chicago, Chicago, Illinois, USA

**Keywords:** osteoblasts, osteoclasts, *Staphylococcus aureus*, RIG-I, cGAS, cytokines, type I interferon

## Abstract

*Staphylococcus aureus* is the primary causative agent of osteomyelitis, and it is now apparent that osteoblasts and osteoclasts play a significant role in the pathogenesis of such infections. Their responses can either be protective or exacerbate inflammatory bone loss and are mediated by the recognition of microbial motifs by various pattern recognition receptors. We have recently reported that osteoblasts can respond to *S. aureus* challenge with the production of the type I interferon, interferon-beta, which can reduce the number of viable bacteria harbored within infected cells. In the present study, we demonstrate that *S. aureus* viability and internalization are necessary for maximal inflammatory cytokine and type I interferon responses of primary bone cells to this pathogen. Importantly, we show that primary murine and human bone cells constitutively express the cytosolic nucleic acid sensors, retinoic acid inducible gene I (RIG-I) and cyclic GMP-AMP synthase (cGAS), and demonstrate that such expression is markedly upregulated following *S. aureus* infection. The functional status of RIG-I and cGAS in osteoblasts and osteoclasts was confirmed by showing that specific ligands for each can also elevate their expression and induce cytokine responses. We have verified the specificity of such responses using siRNA knockdown or pharmacological inhibition and used these approaches to demonstrate that both sensors play a pivotal role in bone cell responses to infection with clinically relevant strains of *S. aureus*. Finally, we have begun to establish the biological significance of RIG-I- and cGAS-mediated bone cell responses with the demonstration that their attenuation increases *S. aureus* burden in infected cells, suggesting a potentially protective role for these sensors in osteomyelitis.

**IMPORTANCE:**

Staphylococcal osteomyelitis is a severe infection that is often recalcitrant to current treatment strategies. We and others have demonstrated that resident bone cells are not merely passive victims but can respond to bacteria with the production of an array of immune mediators, including type I interferons, that could serve to limit such infections. Here, we demonstrate the functional expression of two cytosolic nucleic acid sensors, retinoic acid inducible gene I and cyclic GMP-AMP synthase, in primary murine and human osteoblasts and murine osteoclasts. We show that these pattern recognition receptors mediate potentially protective bone cell type I interferon responses to *Staphylococcus aureus* infection.

## INTRODUCTION

It has become apparent that bone-forming osteoblasts and bone-resorbing osteoclasts have immune functions that play a significant role in the progression of infectious bone diseases, such as staphylococcal osteomyelitis (1–8). Both osteoblasts and osteoclasts express various pattern recognition receptors (PRRs), including membrane-associated Toll-like receptors (TLRs), which enable them to perceive microbial motifs and initiate the production of an array of soluble and cell surface molecules that can exacerbate the inflammatory bone loss commonly associated with bone infection (4, 5, 9–15). However, resident bone cell responses may also serve a protective function against bacterial pathogens, including *Staphylococcus aureus*, that are capable of persisting within resident bone cells (16–21). This notion has recently been supported by our own studies demonstrating that *S. aureus* challenge induces type I interferon (IFN) production by primary osteoblasts that can limit intracellular bacterial burden in infected cells (3, 22). Interestingly, we and others have previously noted that heat or UV-inactivated bacteria are a far weaker stimulus for bone cell responses, suggesting that active internalization and subsequent detection of intracellular bacterial components are essential for maximal immune mediator production (6, 23).

Retinoic acid inducible gene I (RIG-I) and cyclic GMP-AMP synthase (cGAS) have garnered considerable interest as cytosolic sensors for RNA and DNA motifs, respectively, and their critical role in anti-viral immunity in both leukocytic and non-leukocytic cell types has been well documented (24–30). Importantly, these nucleic acid sensors have since been implicated in the perception of intracellular bacteria and the initiation of inflammatory cytokine and type I IFN production by infected cells (31–34). Osteoclasts share a common myeloid lineage with macrophages and dendritic cells and so might be anticipated to express cGAS and RIG-I either constitutively or following activation. In contrast, osteoblasts are of mesenchymal origin but may be capable of expressing these PRRs as evidenced by the expression of mRNA encoding RIG-I in an osteoblastic cell line following TLR3 agonist stimulation (35). Furthermore, we have previously shown that *S. aureus* challenge upregulates the expression of mRNA encoding this cytosolic sensor in primary murine osteoblasts as determined by RNA-Seq analysis (3). In the present study, we have investigated RIG-I and cGAS protein expression in primary osteoblasts and osteoclasts and assessed the relative importance of such expression for responses to *S. aureus* challenge. We demonstrate that osteoblasts and osteoclasts constitutively express these sensors and show that RIG-I and cGAS protein levels are significantly increased in both cell types following *S. aureus* internalization. The functional status of RIG-I and cGAS in osteoblasts and osteoclasts has been confirmed with the demonstration that specific ligands for each can also elevate their expression and induce cytokine responses. We have confirmed the specificity of such responses using siRNA knockdown or pharmacological inhibition and used these approaches to demonstrate that both sensors play a pivotal role in bone cell responses to *S. aureus* infection. Finally, we have begun to establish the biological significance of RIG-I- and cGAS-mediated bone cell responses to infection with the demonstration that their attenuation increases *S. aureus* burden in infected cells, suggesting a potentially protective role for these sensors in the context of osteomyelitis.

## MATERIALS AND METHODS

### Isolation and culture of primary murine osteoblasts

Whole calvaria were isolated from 2- to 3-day-old murine neonates and differentiated for 10 days as previously described (3, 4, 6, 36, 37). Differentiation was confirmed by measuring levels of alkaline phosphatase using a staining kit that is commercially available (Abcam) and microscopy as previously described (3, 36) (Fig. S1A).

### Isolation and culture of bone marrow-derived osteoclasts

Bone marrow was isolated from the tibia and femur of C57BL/6J mice and differentiated to osteoclasts using the receptor activator of nuclear factor kappa-B ligand (RANKL; 100 ng/mL; Sigma, Cat #R0525-10UG) and macrophage colony-stimulating factor (M-CSF; 100 ng/mL; Sigma, Cat #SRP3221-10UG) as previously described (36). Differentiation was confirmed using a commercially available tartrate-resistant acid phosphatase staining kit (Sigma Aldrich) (Fig. S1B).

### *S. aureus* propagation

*S. aureus* strains UAMS1, HFH29568, and TCH1516 were grown to mid-log phase in Luria broth, and the number of colony-forming units was determined using a Genespec3 spectrophotometer as previously described (MiraiBio, Inc.) (3, 4, 36). In some experiments, *S. aureus* was either heat-inactivated for 30 min at 58°C or incubated with fibronectin (5 µg/mL) at room temperature for 30 min prior to infection.

### Pattern recognition receptor ligand transfection of osteoblasts and osteoclasts

Pattern recognition receptor ligands, including B-DNA [poly(dA:dT); VWR; 1 µg/mL], 5′ triphosphate double-stranded RNA (5′ppp dsRNA; InvivoGen; 2 ug/mL), polyinosinic:polycytidylic acid (polyI:C; InvivoGen; 1 µg/mL), and Y-DNA (G3-YSD; InvivoGen; 2.5 µg/mL) were incubated with the lipid-based carrier, Lipofectamine 2000 (L2K; Invitrogen; 7 µg/mL), for 15 min in reduced-serum media. The ligand transfection complexes were then added to osteoblasts and left to incubate for 4 h before the media were replaced with fresh media, absent of transfection reagents. Osteoclasts were transfected in the same manner with B-DNA (0.5 µg/mL), 5′ppp dsRNA (1 µg/mL), or Y-DNA (1 µg/mL). Additionally, bone cells were extracellularly treated with lipopolysaccharide (LPS; Cell Signaling; 10 ng/mL) or PAM3CSK4 (P3C; Invivogen; 10 ng/mL). At the indicated time points, cell supernatants and whole-cell protein isolates were collected for analysis.

### *S. aureus* infection of osteoblasts and osteoclasts

Osteoblasts (1 × 10^6^/well) or osteoclasts (1 × 10^5^/well) were infected with *S. aureus* at varying multiplicities of infection (MOIs) in antibiotic-free media for 2 h as previously described (3, 4, 36). The media were then replaced with media containing 1% penicillin–streptomycin to kill extracellular bacteria. At the indicated time points, cell supernatants and whole-cell protein isolates were collected.

### siRNA transfection of osteoblasts

Osteoblasts were transfected using RNAiMAX according to the manufacturer’s guidelines and as we have previously described (22). The following Silencer Select small interfering RNAs (siRNAs; Thermo Fisher Scientific) were used at a concentration of 15 nM: siRNA targeting RIG-I (assay IDs s106376 and 106374), siRNA targeting RNA polymerase III subunit A (RP3; assay IDs s104375 and s104374), siRNA targeting cGAS (assay IDs s103166 and s103167), or a scrambled, negative control siRNA (catalog numbers 4390846 and 4390844) 24 h prior to transfection with PRR ligands or infection with *S. aureus* as described above. Cell supernatants and whole-cell protein isolates were collected at the indicated time points.

### Enzyme-linked immunosorbent assays

Specific capture enzyme-linked immunosorbent assays (ELISAs) were performed to quantify the production of immune mediators in response to PRR ligands or *S. aureus*. Concentrations of mouse IFN-β and IL-6 were assessed using commercially available antibody pairs (BioLegend and BD BioSciences, respectively) as previously described (3, 36). The recombinant murine proteins for each ELISA were used to generate standard curves. Assessment of the absorbance to the standard curve was used to determine the concentration of each protein of interest in the cell supernatants.

### Immunoblot analysis

Whole-cell lysates were evaluated by a chemiluminescent immunoblot analysis for the expressions of RIG-I, cGAS, and RP3 using a rabbit polyclonal antibody directed against RIG-I (Abcepta, AP1900A), a rabbit monoclonal antibody directed against cGAS (Cell Signaling, clone D3O8O), and a rabbit monoclonal antibody directed against RP3 (Cell Signaling, clone D5Y2D), respectively. The blots were then probed with a mouse monoclonal antibody directed against β-actin (Abcam, catalog no. 49900) to control for differences in protein loading. Densitometric analysis was conducted using ImageLab (BioRad) or Azure Spot Pro Software (Azure Biosystems), and RIG-I, cGAS, and RP3 protein levels were normalized to the expression of β-actin.

### Immunofluorescent microscopy

Osteoblasts were challenged at an MOI of 75:1 with either viable *S. aureus*, bacteria that were heat-inactivated at 58°C for 30 min, or viable bacteria coated with fibronectin (5 µg/mL) for 30 min prior to infection. Cells were fixed at 2 h following bacterial challenge with 4% paraformaldehyde. Extracellular bacteria were tagged first with a solution containing *S. aureus* primary antibody (Invitrogen, PA1-7246) without permeabilization, then an Alexa Fluor 488-conjugated antibody (Invitrogen, A32733). Intracellular bacteria were tagged by a solution containing the same *S. aureus* primary antibody and 0.1% saponin, then an Alexa Fluor 647-conjugated antibody (Invitrogen, A11008). The number of intracellular bacteria was quantified by color thresholding, and assessment of the integrated density was performed using ImageJ (National Institutes of Health, USA).

### Statistical analysis

Data are expressed as the mean ± standard error of the mean (SEM). Commercially available software (GraphPad Prism, GraphPad Software, La Jolla, CA, USA) was used to perform statistical analyses, including Student’s *t*-test, one-way analysis of variance (ANOVA) with Dunnett’s post-hoc test, or two-way ANOVA with Šídák’s multiple comparisons test, as appropriate. For all experiments, a *P* value of < 0.05 was considered statistically significant.

## RESULTS

### *S. aureus* viability and internalization are necessary for maximal stimulation of inflammatory cytokines and type I IFNs by primary murine osteoblasts

**Fig 1:**
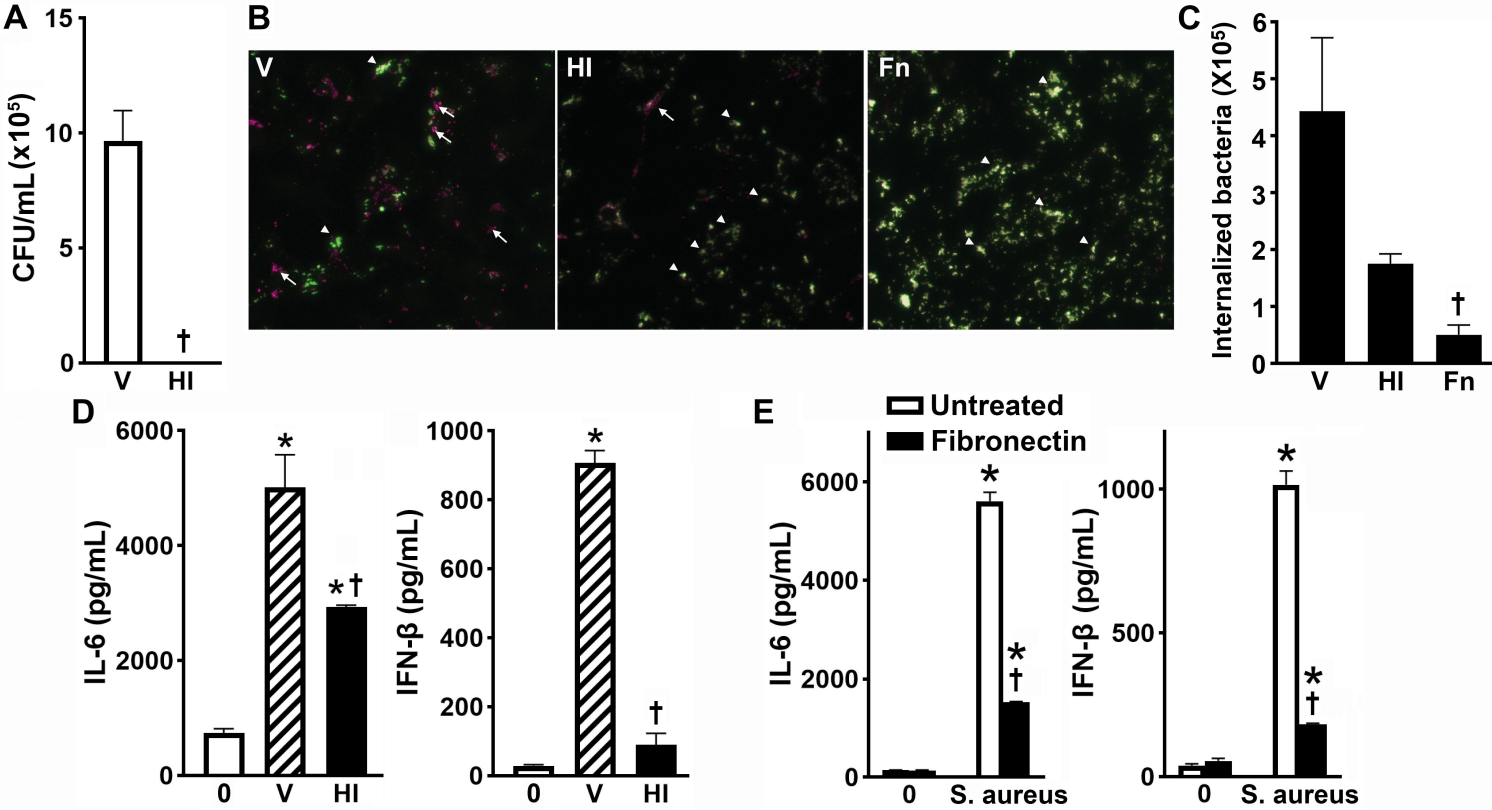
*S. aureus* viability and internalization are necessary for the maximal stimulation of inflammatory cytokine and type I IFN production by primary murine osteoblasts. Osteoblasts were either uninfected (0) or challenged with viable *S. aureus* (V), heat-inactivated bacteria (HI), or viable bacteria coated with fibronectin (Fn; 5 µg/mL) at an MOI of 75:1 for 2 h. Panel A: the number of viable bacteria harbored by osteoblasts challenged with live or heat-inactivated *S. aureus* was assessed by colony counting 8 h post-infection. Panel B: cells were fixed with 4% paraformaldehyde at 2 h following bacterial challenge and processed for immunofluorescent microscopy to label extracellular bacteria (Alexa Fluor 488, green) and intracellular bacteria (Alexa Fluor 647, pink). Arrows indicate intracellular bacteria, while arrowheads indicate extracellular bacteria. Panel C: the number of internalized bacteria was quantified with color thresholding to determine the integrated density using ImageJ at 2 h post-infection. Panel D: at 8 h following viable and inactivated bacterial challenge, osteoblast production of IL-6 and IFN-β was assessed by specific capture ELISA. Panel E: at 8 hours following challenge with uncoated *S. aureus* or fibronectin-coated *S. aureus*, osteoblast production of IL-6 and IFN-β was assessed by specific capture ELISA. Asterisks indicate a statistically significant difference compared to unchallenged osteoblasts. Daggers indicate a statistically significant difference compared to cells challenged with viable bacteria (mean ± SEM, *n* = 3; Student’s *t*-test, *P* value < 0.05).

We first examined the necessity of bacterial viability for the stimulation of IL-6 and IFN-β by osteoblasts (Fig. 1). Osteoblasts were challenged with either viable or heat-inactivated *S. aureus* strain UAMS-1, a clinical osteomyelitis isolate. Consistent with our previous studies (6, 23), viable intracellular *S. aureus* colony-forming units were only recovered following the challenge with viable bacteria and not heat-inactivated *S. aureus* (Fig. 1A). Interestingly, nonviable heat-inactivated *S. aureus* displayed reduced internalization compared to viable *S. aureus* (Fig. 1B and C), and this corresponded with a significantly reduced ability to induce the production of the proinflammatory cytokine, IL-6, and the type I interferon, IFN-β, compared to osteoblasts infected with viable bacteria (Fig. 1D). Interestingly, heat-inactivation reduced, but did not abolish, *S. aureus*-induced IL-6 production but did abolish bacteria-induced IFN-β production.

It is well documented that *S. aureus* fibronectin-binding proteins attach to fibronectin, thereby mediating interactions with osteoblast integrins and driving *S. aureus* internalization (8, 16, 18, 38). Therefore, to confirm that *S. aureus* internalization is necessary for the maximal stimulation of osteoblast proinflammatory and type I interferon responses, internalization was blocked by coating *S. aureus* with fibronectin prior to osteoblast challenge. As shown in Fig. 1B and C, fibronectin-coated *S. aureus* remained extracellular. Importantly, such inhibition of *S. aureus* internalization significantly reduced osteoblast IL-6 and IFN-β production. Together, these data suggest that recognition by intracellular PRRs is required for full stimulation of osteoblast innate immune responses.

### Osteoblasts constitutively express RIG-I and cGAS, and such expression is further increased following infection with *S. aureus*

**Fig 2:**
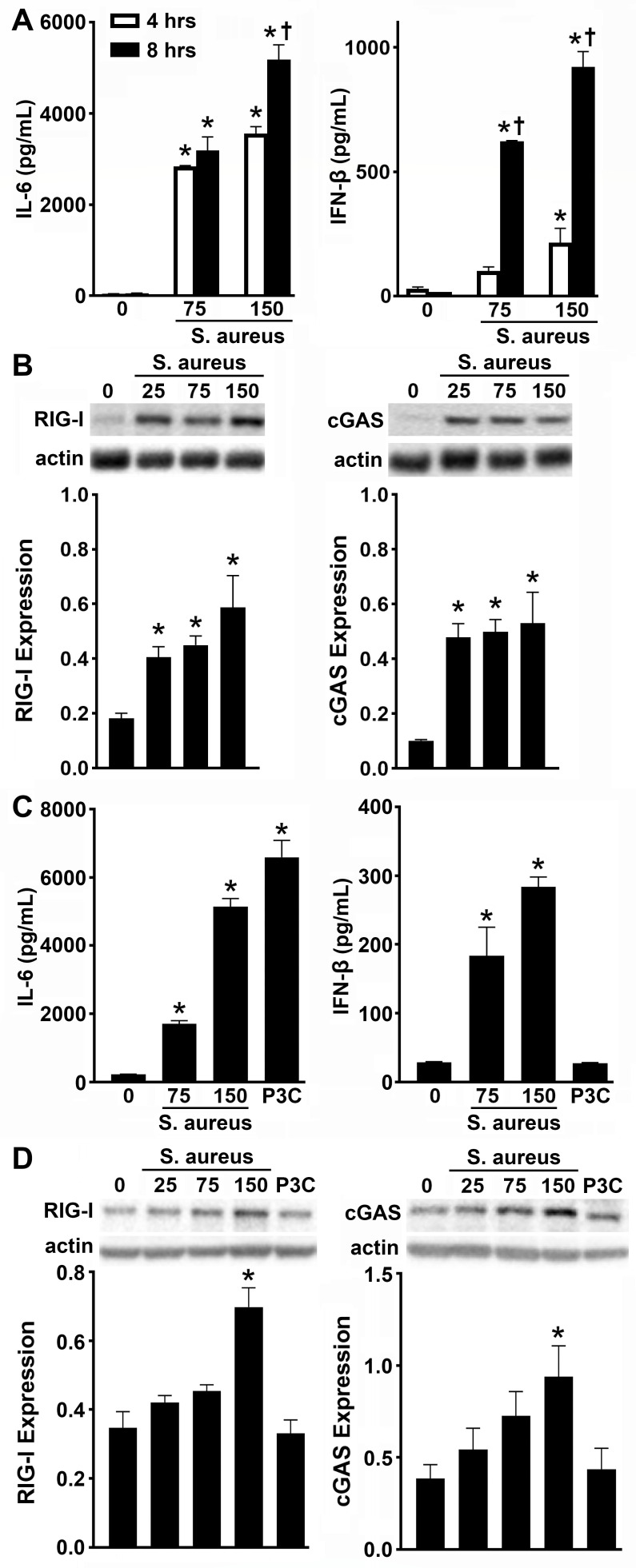
Osteoblasts constitutively express RIG-I and cGAS, and their expression is upregulated following activation associated with *S. aureus* infection. Primary murine (A and B) and primary human (C and D) osteoblasts were untreated (0) or infected with *S. aureus* at MOIs of 25:1, 75:1, or 150:1. Panel A: at 4 and 8 h, IL-6 and IFN-β production was assessed in primary murine osteoblasts by specific capture ELISA. Asterisks indicate a statistically significant difference compared to uninfected osteoblasts. Daggers indicate a statistically significant difference compared to similarly challenged cells at 4 h post-infection (mean ± SEM, *n* = 3; two-way ANOVA, *P* value < 0.05). Panel B: expression of RIG-I (102 kDa) and cGAS (62 kDa) was assessed at 8 h post-infection by immunoblot analysis. Representative immunoblots and the average expression level of each protein, as determined by densitometric analysis normalized to β-actin levels, are shown. Panel C: at 8 h, IL-6 and IFN-β production was assessed in primary human osteoblasts by specific capture ELISA. Panel D: expression of RIG-I and cGAS was assessed by immunoblot analysis. Representative immunoblots and the average expression level of each protein, as determined by densitometric analysis normalized to β-actin levels, are shown. Asterisks indicate a statistically significant difference compared to uninfected osteoblasts (mean ± SEM, *n* = 3; one-way ANOVA or Student’s *t*-test, *P* value < 0.05).

Our published RNA Tag-Seq data indicated a previously unappreciated role for cytosolic nucleic acid sensors in osteoblast responses to *S. aureus* infection (3). Specifically, we reported the enrichment of cytosolic DNA-sensing Kyoto Encyclopedia of Genes and Genomes pathway components, including RNA polymerase III, RIG-I, and TBK1 (3). Therefore, to evaluate the contribution of cytosolic nucleic acid sensors to osteoblast responses to *S. aureu*s, we examined the protein expression of two nucleic acid sensors, RIG-I and cGAS, that converge on the signaling component, TBK1. Consistent with our prior studies (3, 36), *S. aureus* challenge stimulated dose- and time-dependent production of IL-6 and IFN-β by murine osteoblasts (Fig. 2A). Prior to bacterial challenge, osteoblasts displayed low, but demonstrable, constitutive expression of both RIG-I and cGAS (Fig. 2B; Fig. S2A and B). However, concomitant with increases in immune mediator production, RIG-I and cGAS expressions were markedly elevated following *S. aureus* challenge (Fig. 2B; Fig. S2A and B), supporting a role for these sensors during bacterial infection. Consistent with these observations, following *S. aureus* challenge, primary human femoral osteoblasts significantly produced IL-6 and IFN-β (Fig. 2C). As anticipated, the TLR2/TLR1 control ligand, Pam3CSK4, only stimulated IL-6 production by human osteoblasts. Notably, resting human osteoblasts displayed constitutive expression of RIG-I and cGAS, and such expression was only elevated at the highest MOI employed (Fig. 2D; Fig. S2C and D). Together, these data establish that RIG-I and cGAS are expressed by murine and human osteoblasts and support their potential role during *S. aureus* infection.

### Intracellular administration of nucleic acid ligands for RIG-I or cGAS elicits osteoblast immune responses

**Fig 3:**
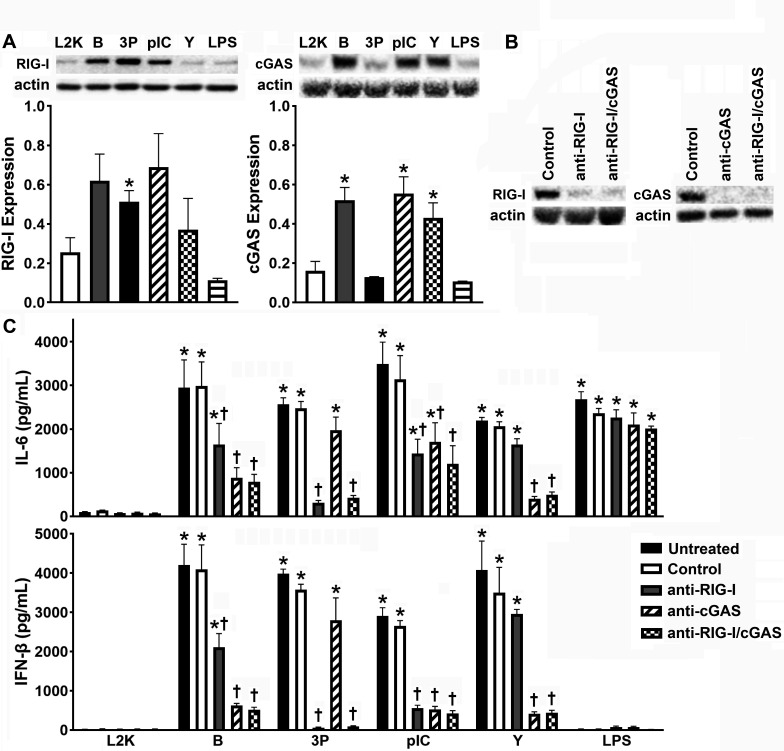
Intracellular administration of nucleic acid ligands for RIG-I or cGAS elicits murine osteoblast immune responses. Panel A: osteoblasts were transfected with B-DNA (B; 1 µg/mL), 5′ triphosphate double-stranded RNA (3P; 2 µg/mL), polyinosinic–polycytidylic acid (pIC; 1 µg/mL), or Y-DNA (Y; 2.5 µg/mL) complexed with Lipofectamine 2000 (L2K) or were treated with L2K alone or challenged with lipopolysaccharide (LPS; 10 ng/mL). At 8 h post-transfection, expression of RIG-I (102 kDa) and cGAS (62 kDa) was assessed by immunoblot analysis. Representative immunoblots and the average expression level of each protein, as determined by densitometric analysis normalized to β-actin levels, are shown. Asterisks indicate a statistically significant difference compared to osteoblasts treated with L2K alone (mean ± SEM, *n* = 2–4; Student’s *t*-test, *P* value < 0.05). Panel B: osteoblasts were transfected with siRNA (15 nM) directed against RIG-I, cGAS, or both using RNAiMAX or exposed to control siRNA (Control). At 8 h, RIG-I and cGAS expressions were assessed by immunoblot analysis. Panel C: cells were transfected with siRNA (15 nM) directed against RIG-I, cGAS, both, or control siRNA prior to intracellular challenge with B-DNA (B; 1 µg/mL), 5′ triphosphate double stranded RNA (3P; 2 µg/mL), polyinosinic−polycytidylic acid (pIC; 1 µg/mL), or Y-DNA (Y; 2.5 µg/mL) complexed with L2K or were treated with L2K alone or challenged with LPS (10 ng/mL). At 8 h, production of IL-6 and IFN-β was assessed with specific capture ELISA. Asterisks indicate significance compared to the L2K treatment alone. Daggers represent significance compared to the similarly stimulated control siRNA-treated group (mean ± SEM, *n* = 3–6; two-way ANOVA with Šídák’s multiple comparison test, *P* value < 0.05).

To further characterize RIG-I and cGAS expressions in primary murine osteoblasts and begin to establish the functional nature of such expression, we employed documented ligands for these cytosolic nucleic acid sensors. We challenged osteoblasts with intracellular administration of nonspecific DNA and RNA ligands, B-DNA and polyI:C, and the RIG-I and cGAS specific ligands, 5′ppp RNA and Y-DNA (G3-YSD), respectively. As shown in Fig. 3A, RIG-I and cGAS expressions were significantly elevated following challenge with either B-DNA or polyI:C from the low-level constitutive expression of each in unstimulated cells (Fig. S3A and B). Interestingly, treatment with the cGAS and RIG-I specific ligands only upregulated the expression of their own receptor. In contrast, our negative control LPS, a known TLR4 ligand, failed to impact the expression of either RIG-I or cGAS by osteoblasts. Together, these data indicate that osteoblasts are responsive to RIG-I and cGAS ligands and further support the notion that the expression of these sensors can be upregulated following activation.

Next, to assess the contribution of each of these sensors in osteoblast responses to intracellular nucleic acids, we employed an siRNA approach to knock down expressions of RIG-I and cGAS. While the administration of negative control siRNA alone upregulated the expressions of both RIG-I and cGAS, the introduction of siRNA targeting RIG-I and/or cGAS knocked down the expression of these sensors with high efficiency (Fig. 3B; Fig. S3C and D). In agreement with studies in other cell types (26, 29, 30, 34, 39–42), delivery of the RNA ligands, 5′ppp RNA and polyI:C, and the DNA ligands, B-DNA and Y-DNA, stimulated significant production of both IL-6 and IFN-β by osteoblasts (Fig. 3C). RIG-I knockdown significantly reduced 5′ppp RNA and polyI:C induced IL-6 and IFN-β production by osteoblasts, consistent with the detection of RNA motifs by this sensor. Interestingly, RIG-I knockdown also significantly reduced responses to B-DNA (Fig. 3C). This effect is consistent with the established ability of RIG-I to indirectly identify B-DNA via an RNA polymerase III-dependent mechanism (26, 40). Additionally, we observed that cGAS knockdown significantly reduced IL-6 and IFN-β production by osteoblasts following stimulation with B-DNA, Y-DNA, and polyI:C (Fig. 3C), consistent with data in other cell types (29, 30, 34, 41–43). As anticipated, the TLR4 control ligand, LPS, stimulated IL-6 production by osteoblasts but failed to elicit IFN-β release, and IL-6 production was not affected by either RIG-I or cGAS knockdown (Fig. 3C).

Collectively, these data further support the constitutive and inducible expression of RIG-I and cGAS by primary osteoblasts and establish the functional status of such expression with the demonstration that these sensors mediate osteoblast inflammatory and type I IFN responses to foreign nucleic acids.

### RIG-I and cGAS mediate the inflammatory and anti-microbial IFN responses of osteoblasts to *S. aureus* infection

**Fig 4:**
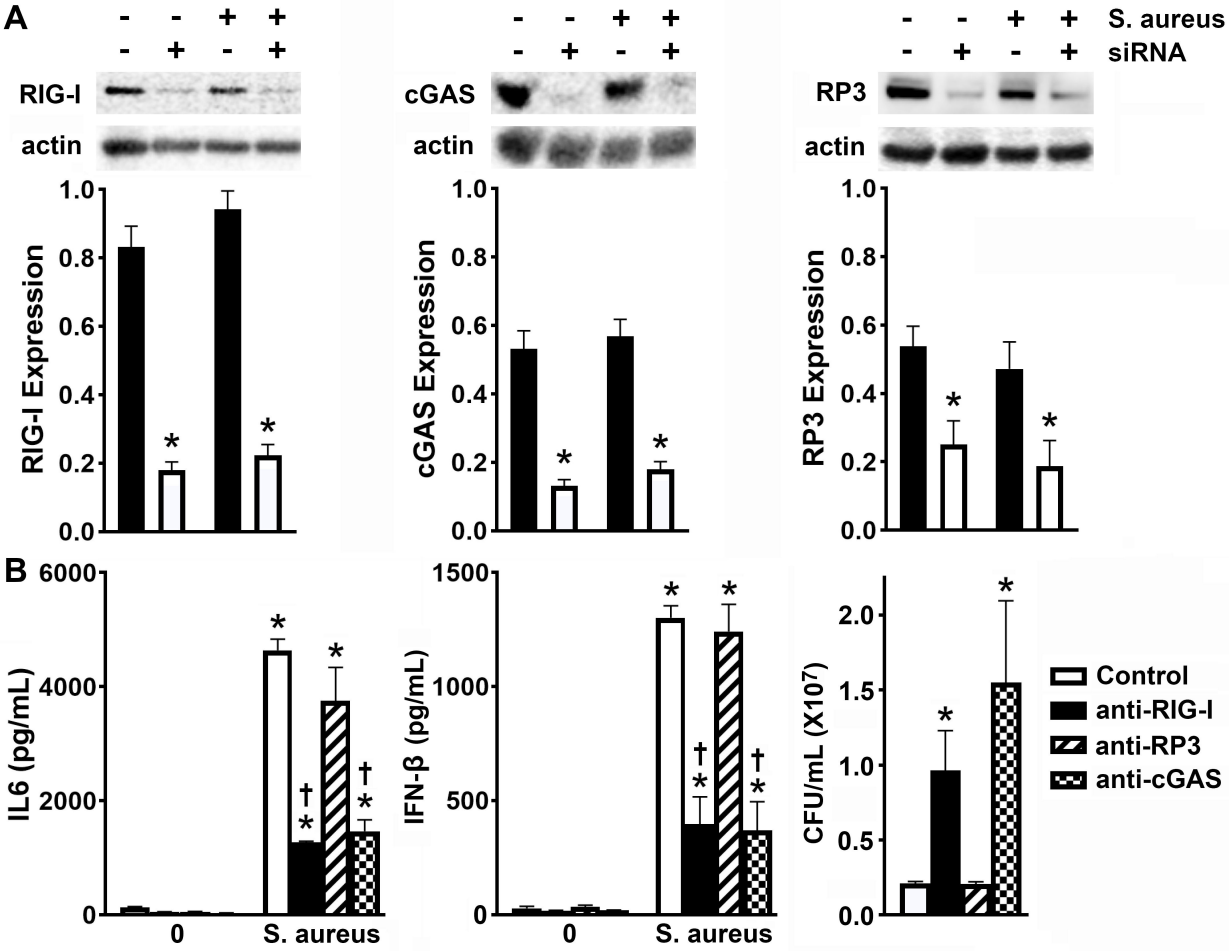
RIG-I and cGAS mediate the inflammatory and anti-microbial IFN responses of murine osteoblasts to *S. aureus* infection. Osteoblasts were transfected with siRNA (10 nM) directed against RIG-I, cGAS, or RNA polymerase III (RP3) or control siRNA (Control) using RNAiMAX. These cells were then uninfected (0) or challenged with *S. aureus* (MOI of 75:1). At 8 h post-infection, RIG-I (102 kDa), cGAS (62 kDa), and RP3 (165 kDa) expressions were quantified by immunoblot analysis (A). Representative immunoblots and the average expression level of each protein, as determined by densitometric analysis normalized to β-actin levels, are shown. Asterisks indicate a significant decrease compared to the corresponding control siRNA-treated group (mean ± SEM, *n* = 3–6; one-way ANOVA, *P* value < 0.05). In addition, IL-6 and IFN-β production by these cells was assessed by specific capture ELISA, and intracellular bacterial viability was assessed by colony counting 24 h post-infection (B). Asterisks indicate a significant increase compared to uninfected cells. Daggers represent a significant decrease compared to the similarly infected control siRNA-treated group (mean ± SEM, *n* = 3–6; two-way ANOVA with Šídák’s multiple comparison test and Student’s *t*-test, *P* value < 0.05).

To evaluate the biological role of osteoblast cytosolic nucleic acid sensors during *S. aureus* challenge, we again employed siRNA approaches to knock down the expression of RIG-I and cGAS (Fig. 4A; Fig. S4A and B). As shown in Fig. 4B, *S. aureus-*infected osteoblasts displayed significantly less IL-6 and IFN-β production following RIG-I knockdown. RIG-I cannot only identify 5′ppp RNA directly but also detect B-DNA indirectly via RNA polymerase III activity (26, 40). Therefore, to determine if RIG-I activation is due to the detection of *S. aureus* RNA or DNA, we knocked down expression of the RNA polymerase III (RP3) catalytic subunit (Fig. 4A; Fig. S4C). As shown in Fig. 4B, RP3 knockdown had no significant effect on IL-6 or IFN-β production by infected osteoblasts, suggesting that RIG-I is activated by bacterial RNA. Additionally, we examined the contribution made by cGAS to osteoblast responses to *S. aureus*. Following cGAS knockdown, osteoblasts produced significantly less IL-6 and IFN-β following challenge (Fig. 4B). These data suggest that the inflammatory and type I IFN responses of *S. aureus*-infected osteoblasts are mediated by the detection of bacterial RNA and DNA that occurs via RIG-I and cGAS, respectively.

Our recent studies have indicated a role for type I IFNs in restricting intracellular bacterial burden in *S. aureus-*infected osteoblasts (3, 22), and so we evaluated the effect of RIG-I and cGAS knockdown on the number of viable bacteria harbored by infected cells. Consistent with their observed effects on IFN-β production, RIG-I and cGAS knockdown, but not RP3 knockdown, both led to a significant increase in intracellular bacterial burden compared to the negative control, suggesting that RIG-I- and cGAS-mediated detection of bacterial RNA and DNA, respectively, contributes to restriction of bacterial burden inside osteoblasts (Fig. 4B).

**Fig 5:**
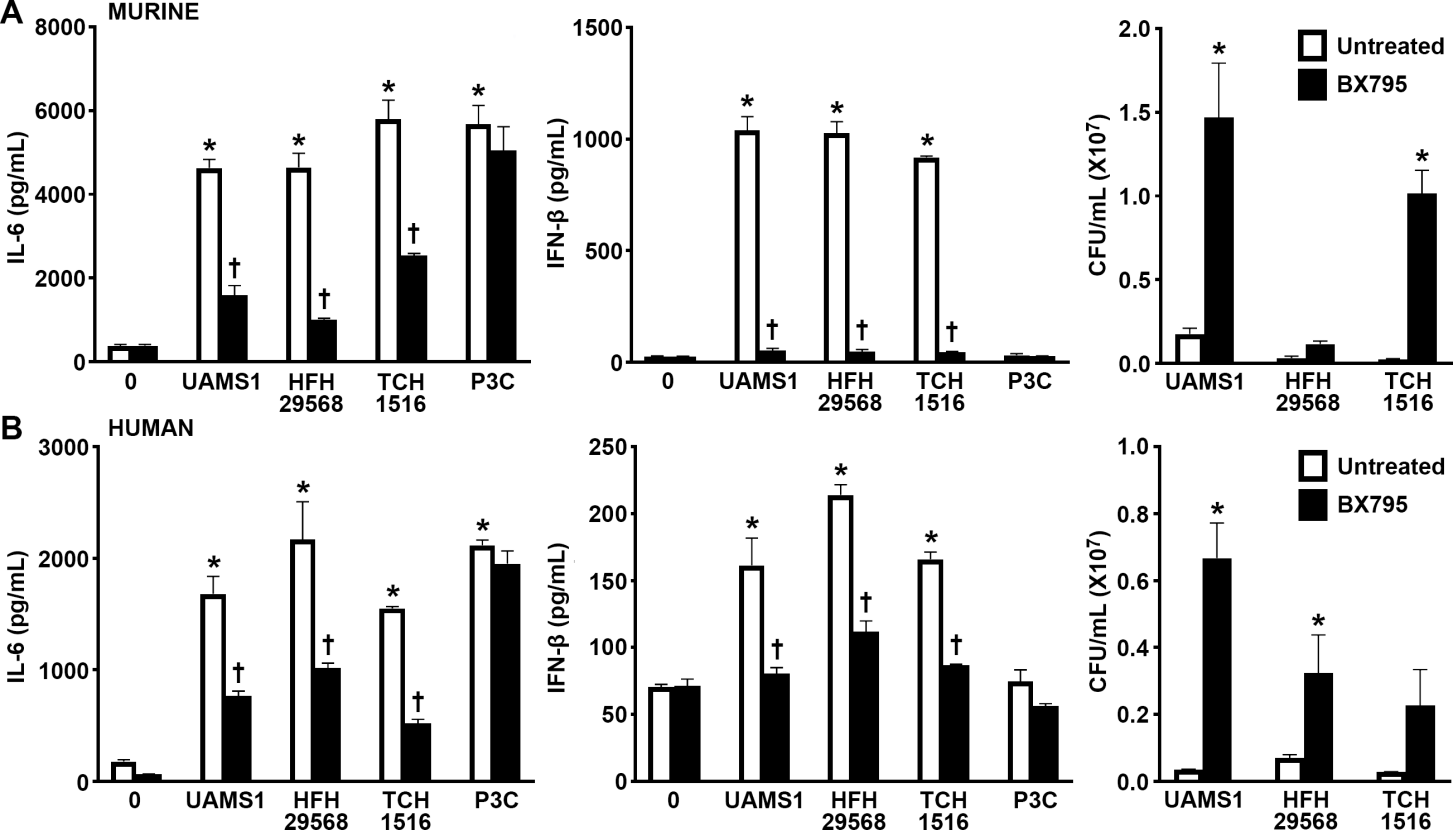
RIG-I and cGAS mediate the inflammatory and anti-microbial IFN responses of primary murine and human osteoblasts to challenge with clinically relevant *S. aureus* strains. Primary murine (A) and human (B) were either untreated or treated with a TBK1/IKKε inhibitor (BX795; 2 µM) for 4 h prior to challenge with *S. aureus* strains (MOI of 75:1) UAMS1, HFH29568, and TCH 1516 or Pam3CSK4 (P3C: 10 ng/mL). At 8 h post-infection, IL-6 and IFN-β production was assessed with specific capture ELISAs. Intracellular bacterial viability was assessed by colony counting at 24 h post-infection. Asterisks indicate significance compared to uninfected cells. Daggers indicate significance compared to the similarly infected but untreated group (mean ± SEM, *n* = 3; two-way ANOVA with Šídák’s multiple comparison test, *P* value < 0.05).

To further support these observations, primary murine and human femoral osteoblasts were challenged with three clinically relevant strains of *S. aureus* in the absence and presence of a pharmacological inhibitor of TBK1/Ikkε, a common RIG-I and cGAS downstream signaling component. Similar to the clinical osteomyelitis *S. aureus* strain UAMS-1, the USA300 methicillin-resistant strains, HFH 29568 and TCH 1516, stimulated a significant production of IL-6 and IFN-β by primary murine and human osteoblasts (Fig. 5A and B). Treatment with the TBK1/Ikkε inhibitor, BX795, significantly reduced the production of both IL-6 and IFN-β by *S. aureus-*infected murine and human osteoblasts (Fig. 5A and B). As anticipated, our control TLR2/TLR1 ligand, Pam3CSK4, stimulated only osteoblast production of IL-6, and such production was not impacted by BX795 treatment (Fig. 5). Notably, inhibition of RIG-I and cGAS downstream signaling led to increased intracellular bacterial burden compared to the untreated control (Fig. 5), indicating that RIG-I- and cGAS-mediated responses contribute to the restriction of bacterial burden inside murine and human osteoblasts.

### Activated osteoclasts express RIG-I and cGAS, and these sensors similarly mediate the antimicrobial IFN responses of these cells to *S. aureus* infection

**Fig 6:**
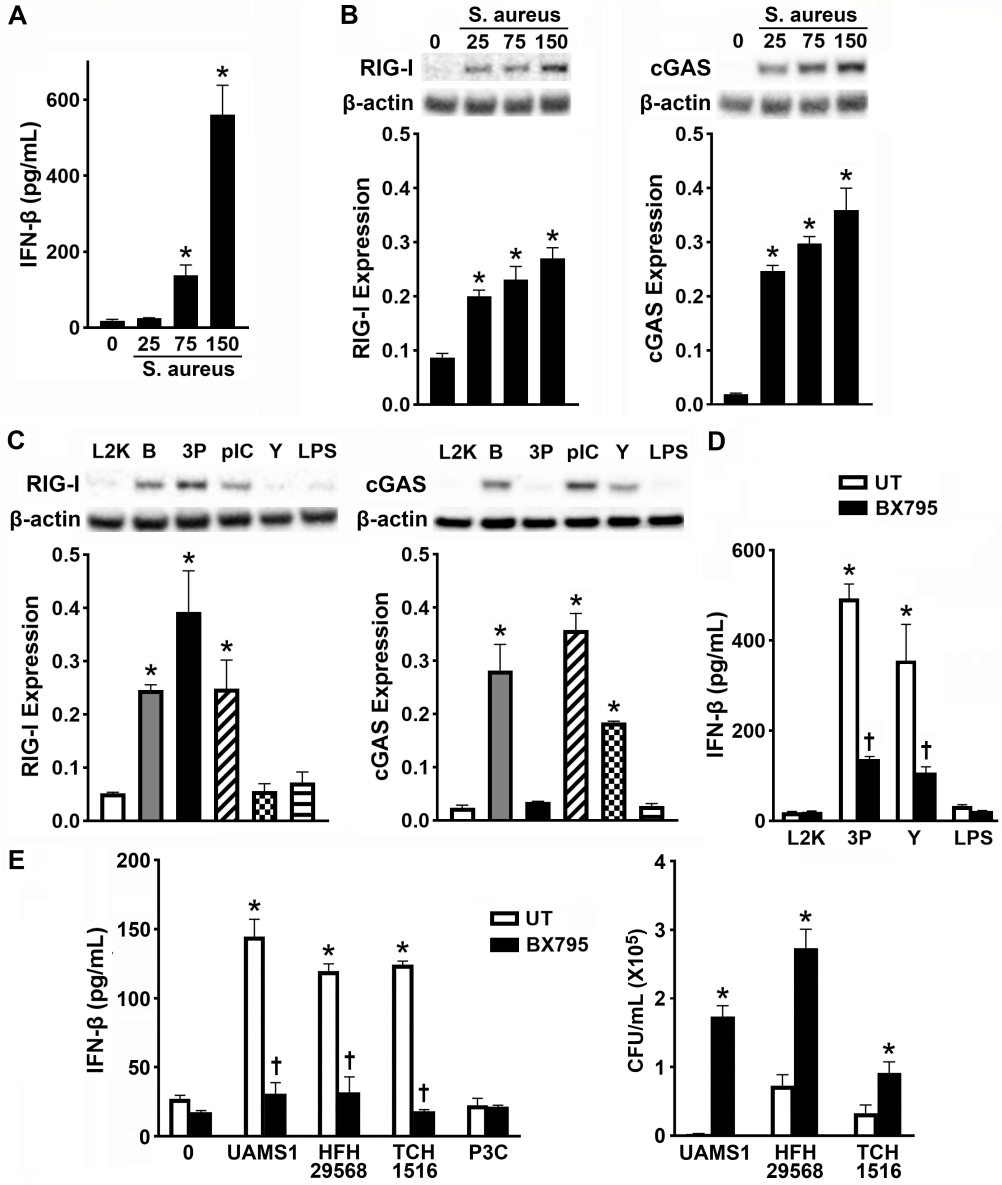
Activated osteoclasts express RIG-I and cGAS, and these sensors similarly mediate the anti-microbial IFN responses of these cells to *S. aureus* infection. Panels A and B: bone marrow-derived primary murine osteoclasts were untreated (0) or infected with *S. aureus* at MOIs of 25:1, 75:1, or 150:1. At 8 h, production of IFN-β was determined by specific capture ELISA (A), and expressions of RIG-I (102 kDa) and cGAS (62 kDa) were assessed by immunoblot analysis (B). Representative immunoblots and the average expression level of each protein, as determined by densitometric analysis normalized to β-actin levels, are shown. Asterisks indicate a significant increase compared to unchallenged cells. Panel C: osteoclasts were transfected with B-DNA (B; 0.5 µg/mL), 5′ triphosphate double-stranded RNA (3P; 1 µg/mL), polyinosinic–polycytidylic acid (pIC; 0.5 µg/mL), or Y-DNA (Y; 1 µg/mL) complexed with L2K or were treated with L2K alone or challenged with LPS (5 ng/mL). At 8 h post-transfection, expressions of RIG-I and cGAS were assessed by immunoblot analysis. Representative immunoblots and the average expression level of each, as determined by densitometric analysis normalized to β-actin levels, are shown. Asterisks indicate a statistically significant difference compared to osteoclasts treated with L2K alone (mean ± SEM, *n* = 3–4; Student’s *t*-test, *P* value < 0.05). Panels D and E: osteoclasts were either untreated or treated with a TBK1/IKKε inhibitor (BX795; 1 µM) for 2 h prior to challenge with RIG-I or cGAS nucleic acid agonists, *S. aureus* infection (MOI of 75), or Pam3CSK4 (P3C: 10 ng/mL). At 8 h, IFN-β production was assessed by specific capture ELISA, and viable intracellular bacterial burden was assessed at 24 h post-infection by colony counting. Asterisks indicate a significance compared to unchallenged cells. Daggers represent significance compared to the corresponding untreated group (mean ± SEM, *n* = 3; two-way ANOVA with Šídák’s multiple comparison test and Student’s *t*-test, *P* value < 0.05).

Similar to osteoblasts, bone-resorbing osteoclasts express PRRs that can initiate innate immune responses to *S. aureus* infection in bone tissue (10, 13, 14, 44). Here, we report that primary murine bone marrow-derived osteoclasts produced IFN-β in a dose-dependent manner following *S. aureus* challenge (Fig. 6A). As shown in Fig. 6B, while osteoclasts display minimal constitutive protein expressions of RIG-I and cGAS, the levels of both were significantly upregulated following infection with *S. aureus* strain UAMS-1 in a dose-dependent manner (Fig. S5).

To assess the functionality of RIG-I and cGAS in osteoclasts, we again employed specific and non-specific nucleic acid ligands for these sensors. Consistent with our findings in osteoblasts, osteoclast RIG-I expression was markedly increased following intracellular administration of the RNA ligands 5′ppp RNA and polyI:C and also the non-specific DNA ligand, B-DNA, but not cGAS-specific Y-DNA or the TLR4 ligand LPS (Fig. 6C; Fig. S5). Similarly, we observed increased expression of cGAS following stimulation with the DNA ligands, B-DNA and Y-DNA, and the non-specific RNA, polyI:C, but not RIG-I-specific 5′pppRNA or the TLR4 ligand LPS (Fig. 6C; Fig. S5).

Importantly, the RIG-I- and cGAS-specific ligands, 5′ppp RNA and Y-DNA, respectively, stimulated the production of IL-6 and IFN-β by osteoclasts that was significantly reduced following pharmacological inhibition of TBK1/Ikkε, a common RIG-I and cGAS downstream signaling component (Fig. 6D). This supports a role for RIG-I and cGAS in the initiation of osteoclast type I IFN responses to foreign RNA and DNA, respectively. As such, we next evaluated the contribution of RIG-I and cGAS to *S. aureus*-mediated osteoclast IFN-β responses. As shown in Fig. 6E, infection with the three clinically relevant strains, UAMS-1, HFH 29568, and TCH 1516, stimulated significant IFN-β production by osteoclasts, and such production was significantly reduced following TBK1/Ikkε inhibition. Furthermore, inhibition of RIG-I and cGAS downstream signaling resulted in a significant increase in the bacterial burden in infected cells, supporting a protective role for these sensors in osteoclasts (Fig. 6E).

## DISCUSSION

Staphylococcal osteomyelitis causes significant morbidity due to severe inflammation and disruption of bone homeostasis leading to net bone loss (45–48). It is well established that resident bone cells contribute to the production of inflammatory immune mediators during infection via recognition of *S. aureus* by PRRs, including Toll- and NOD-like receptors (2, 4, 5, 10, 12–14, 44, 49). Consistent with previous studies (6, 23), we demonstrate that bacterial viability is necessary for maximal immune stimulation, including the production of the inflammatory cytokine, IL-6, and the production of the type I IFN, IFN-β. We further show that the reduced responses to heat-inactivated *S. aureus* can be attributed to reduced bacterial internalization into osteoblasts. While *S. aureus* is considered an extracellular pathogen, *S. aureus* can attach to and invade resident bone cells, including osteoblasts, osteocytes, and osteoclasts (8, 18, 20, 21, 50). Here, we demonstrate that blocking bacterial internalization dramatically reduces IL-6 release and almost totally abolishes IFN-β production by osteoblasts, supporting the necessity of internalization for the full stimulation of osteoblast responses. This requirement suggests a key role for intracellular PRRs in the stimulation of osteoblast responses to *S. aureus* infection.

Our published RNA Tag-Seq analyses indicate an unappreciated role for intracellular nucleic acid sensing pathway components, including the cytosolic sensors RIG-I and cGAS, in osteoblast responses to *S. aureus* infection (3). Additionally, previous studies have demonstrated the expression of RIG-I in fibroblasts and the murine osteoblast cell line, MC3T3-E, at the level of mRNA, and the expression of cGAS in human osteosarcoma cells (35, 51, 52). Such RIG-I and cGAS expressions were found to be upregulated following polyI:C challenge and radiation-induced DNA damage, respectively (35, 51, 52). Furthermore, mesenchymal stem cells, the progenitor cells for osteoblasts, have been reported to express RIG-I (53). In the present study, we observed that primary murine osteoblasts express RIG-I and cGAS protein at low, but demonstrable, levels at rest and are markedly upregulated following intracellular delivery of foreign nucleic acids. Interestingly, such upregulation appears to show specificity as RIG-I and cGAS specific ligands each induced the expression of their corresponding receptor but failed to induce the expression of the other, while the TLR4 ligand, LPS, failed to upregulate the expression of either sensor. This inducibility implies functionality, and this is further supported by the ability of these nucleic acid ligands to induce inflammatory cytokine and type I IFN production by osteoblasts. However, more definitive proof of the functional expression of both RIG-I and cGAS by osteoblasts comes from the demonstration that these sensors are required for nucleic acid-induced osteoblast production of these immune mediators, as demonstrated by the sensitivity of these responses to siRNA knockdown and pharmacological inhibition.

Similar to osteoblasts, we found that primary murine osteoclasts derived from bone marrow hematopoietic progenitor cells express RIG-I and cGAS and showed that such expression was also increased in a specific manner following the delivery of nucleic acid ligands for each. Such a finding might not be considered surprising due to their myeloid lineage, but it is noteworthy that these cells express very low levels of these sensor proteins prior to stimulation. Importantly, we established that RIG-I and cGAS are functional in osteoclasts, with the demonstration that specific ligands for each stimulate type I IFN production by these cells and confirmed that such responses were mediated via these sensors by showing their abolition following siRNA knockdown.

The biological significance of RIG-I and cGAS expressions in bone cells is suggested by the upregulated expression of both of these cytosolic sensors following *S. aureus* infection of osteoblasts and osteoclasts. More direct evidence for a key role for RIG-I and cGAS in bone cell responses comes from our demonstration that these sensors are required for maximal osteoblast and osteoclast immune responses following *S. aureus* challenge. We have previously demonstrated that IFN-β is produced in bone tissue in a murine model of staphylococcal osteomyelitis (3, 36) and showed that the clinical *S. aureus* osteomyelitis isolate, UAMS-1, stimulates dose-dependent production of IFN-β by primary murine osteoblasts (3). In the present study, we demonstrate that primary murine and human osteoblasts significantly produce IFN-β following challenge with UAMS-1 or HFH 29568 and TCH 1516, two USA300 methicillin-resistant strains. Similarly, osteoclasts also produce this type I IFN following *S. aureus* infection. Importantly, RIG-I and cGAS knockdown or pharmacological inhibition of their downstream signaling attenuates inflammatory cytokine release and abolishes IFN-β production by osteoblasts and osteoclasts. Notably, following internalization, *S. aureus* is contained initially within an endosome compartment but can later escape to the cytosol. As such, residual cytokine production may be attributable to other PRRs, including the endosomal receptor, TLR9, or the cytosolic sensor, NOD2, which have previously been documented to contribute to bone cell responses to *S. aureus* challenge (2, 5, 9, 10). Furthermore, our data support the notion that *S. aureus* RNA is recognized by RIG-I, while bacterial DNA is exclusively recognized by cGAS despite the known ability of RIG-I to indirectly identify DNA ligands via an RNA polymerase III-dependent mechanism. However, we acknowledge that additional studies will be required to conclusively demonstrate that *S. aureus* RNA and DNA are accessible in the cytosol and directly recognized by RIG-I and cGAS, respectively.

While the contribution made by RIG-I- and cGAS-mediated osteoblast and osteoclast immune responses to osteomyelitis pathogenesis has yet to be determined, it is possible that the induced production of inflammatory/pro-osteoclastogenic cytokines, such as IL-6, exacerbates the inflammatory bone loss associated with *S. aureus* infection. Alternatively, RIG-I- and/or cGAS-induced IFN-β production may serve a protective role given our previous studies demonstrating the ability of this type I IFN to reduce intracellular bacterial burden in *S. aureus-*infected osteoblasts and osteoclasts (3, 22). This notion is supported by the present demonstration that RIG-I and cGAS expression knockdown or inhibition of their downstream signaling pathways and the subsequent abolition of *S. aureus*-induced IFN-β production are associated with a significant increase in the number of viable bacteria harbored in infected osteoblasts and osteoclasts.

Collectively, these studies support a model in which viable *S. aureus* is internalized by resident bone cells to stimulate maximal inflammatory and type I IFN responses. Such responses are dependent on the activation of the cytosolic nucleic acid sensors, RIG-I and cGAS, which are upregulated following bacterial challenge. Specifically, RIG-I and cGAS identify *S. aureus* RNA and DNA, respectively, leading to the production of proinflammatory cytokines and the type I IFN, IFN-β, by infected osteoblasts and osteoclasts. While such responses might contribute to inflammatory bone loss, RIG-I- and cGAS-dependent type I IFN production may serve a protective role during osteomyelitis by restricting *S. aureus* survival and/or replication in infected osteoblasts and osteoclasts.

## Data Availability

The authors confirm that the data supporting the findings of this study are available within the article and/or its supplemental materials. Any additional data that support the findings of this study are available from the corresponding author upon reasonable request.

## SUPPLEMENTAL MATERIAL

Supplemental Figures mbio.03971-24-s0001.docx Figures S1 to S5.

ASM does not own the copyrights to Supplemental Material that may be linked to, or accessed through, an article. The authors have granted ASM a non-exclusive, world-wide license to publish the Supplemental Material files. Please contact the corresponding author directly for reuse.
