# Supplementary material for: RIG-I and cGAS mediate antimicrobial and inflammatory responses of primary osteoblasts and osteoclasts to Staphylococcus aureus
Source: mBio. 2025 Mar 26;16(5):e03971-24. doi: 10.1128/mbio.03971-24 (PMC12077190; doi:10.1128/mbio.03971-24)
Supplement: Supplemental Figures — Figures S1 to S5. [file mbio.03971-24-s0001.docx]

**Supporting information for**

**RIG-I and cGAS mediate antimicrobial and inflammatory responses of primary osteoblasts and osteoclasts to *Staphylococcus aureus***

Erin L. Mills^1^, Samantha R. Suptela^1^, Mary-Kate Key^2^, Ian Marriott^1^, and M. Brittany Johnson^1^

^1^Department of Biological Sciences, University of North Carolina at Charlotte, Charlotte, NC, USA, 28223

^2^Graduate Division of Biological and Biomedical Sciences, Emory University, Atlanta, GA, USA, 30322

**SUPPORTING INFORMATION**

**
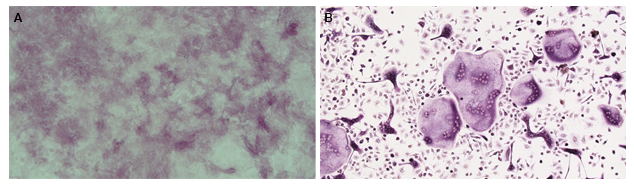
**

**Supplemental Figure 1:** Panel A: Primary murine osteoblasts were differentiated for 10 days in media containing 0.1 M ascorbic acid and 1 M β-glycerophosphate. After 10 days cells were fixed with 4% paraformaldehyde (PFA) and stained for the presence of alkaline phosphatase (ALP). Panel B: Primary murine osteoclasts were differentiated for 5 days in media containing receptor activator of nuclear factor kappa-Β ligand (RANKL; 100 ng/mL) and macrophage colony-stimulating factor (M-CSF; 100 ng/mL). After 5 days cells were fixed with 4% paraformaldehyde (PFA) and tartrate-resistant acid phosphatase (TRAP) staining was performed.

**
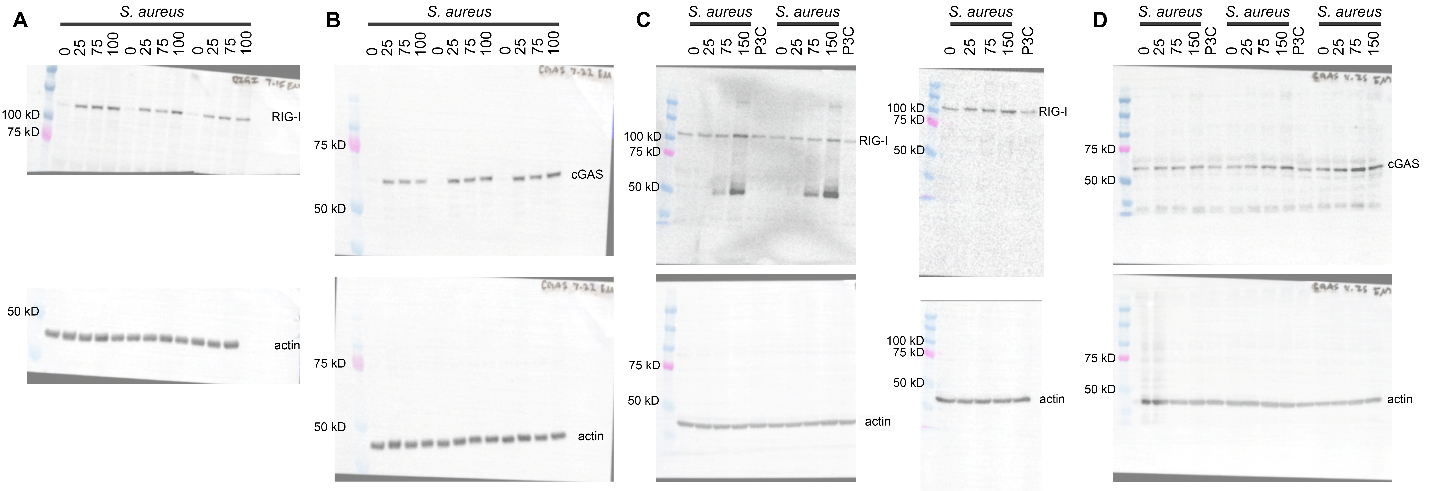
**

**Supplemental Figure 2:** Primary murine (Panel A-B) and human (Panel C-D) osteoblasts were untreated (0) or infected with *S. aureus* at MOIs of 25:1, 75:1, or 150:1. At 8 hours, expression of RIG-I (102 kDa) and cGAS (62 kDa) was assessed by immunoblot analysis and normalized to β-actin levels and representative immunoblots are shown.


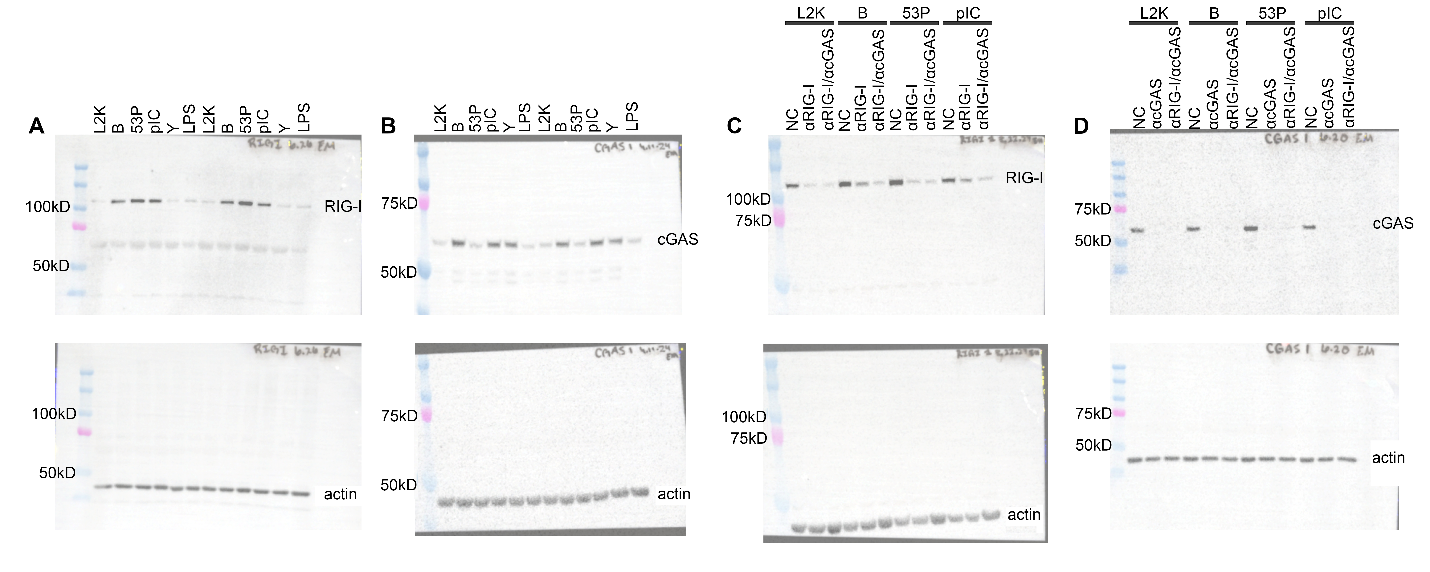


**Supplemental Figure 3:** Osteoblasts were transfected with B-DNA (B; 1 µg/mL), 5' triphosphate double stranded RNA (53P; 2 µg/mL), polyinosinic-polycytidylic acid (pIC; 1 µg/mL), or Y-DNA (Y; 2.5 µg/mL) complexed with lipofectamine 2000 (L2K), or were treated with L2K alone or challenged with lipopolysaccharide (LPS; 10 ng/mL).  At 8 hours post-transfection, expression of RIG-I (102 kDa) and cGAS (62 kDa) was assessed by immunoblot analysis and normalized to β-actin levels and representative immunoblots are shown (A-B). Cells were transfected with siRNA (15 nM) directed against RIG-I, cGAS, both, or control siRNA, prior to intracellular challenge with B-form DNA (B; 1 µg/mL), 5' triphosphate double stranded RNA (53P; 2 µg/mL), polyinosinic-polycytidylic acid (pIC; 1 µg/mL), or Y-form DNA (Y; 2.5 µg/mL) complexed with L2K, or were treated with L2K alone or challenged with LPS (10 ng/mL). At 8 hours post-transfection, expression of RIG-I (102 kDa) and cGAS (62 kDa) was assessed by immunoblot analysis and normalized to β-actin levels and representative immunoblots are shown (C-D).


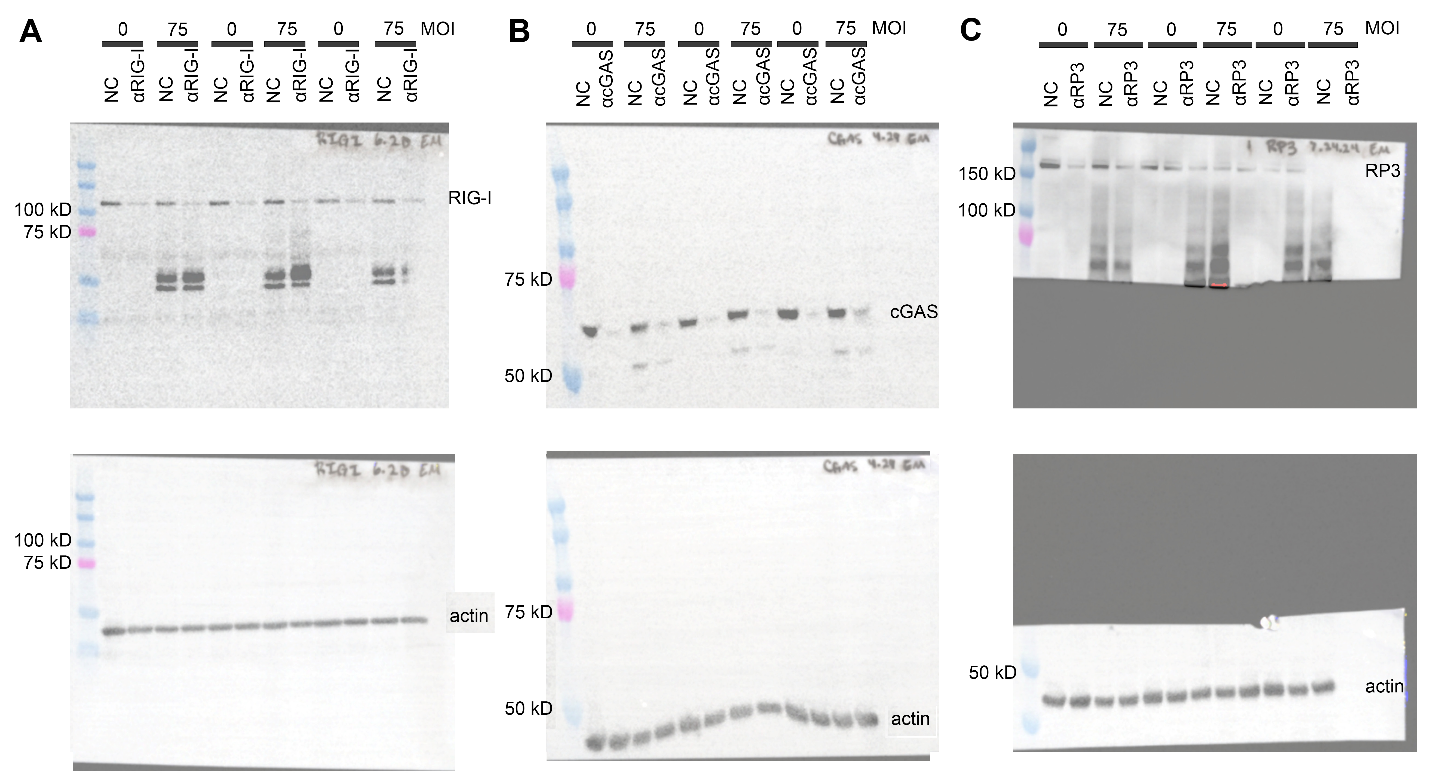


**Supplemental Figure 4:** Osteoblasts were transfected with siRNA (10 nM) directed against RIG-I, cGAS, or RNA polymerase III (RP3), or control siRNA (Control) using RNAiMAX.  These cells were then uninfected (0) or challenged with *S. aureus* (MOI of 75:1).  At 8 hours post-infection, RIG-I (102 kDa), cGAS (62 kDa), and RP3 (165 kDa) expression was quantified by immunoblot analysis and normalized to β-actin levels and representative immunoblots are shown (A-C).


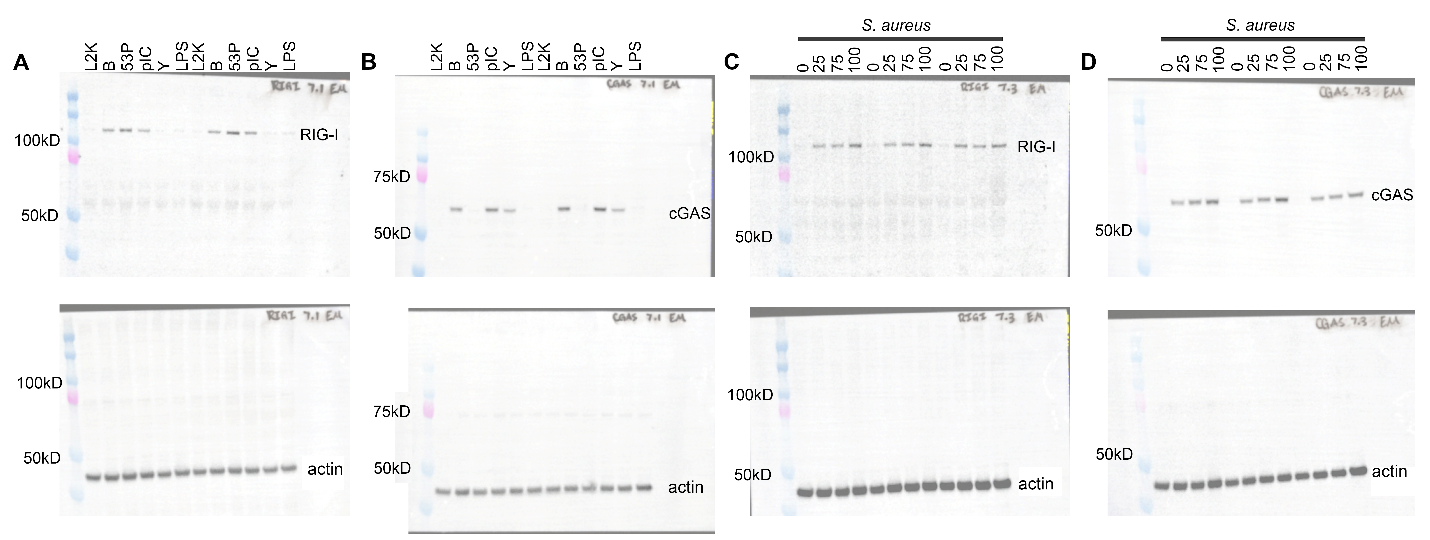


**Supplemental Figure 5:** Bone marrow-derived primary murine osteoclasts were untreated (0) or infected with *S. aureus* at MOIs of 25:1, 75:1, or 150:1. At 8 hours, expression of RIG-I (102 kDa) and cGAS (62 kDa) was assessed by immunoblot analysis and normalized to β-actin levels and representative immunoblots are shown (C-D). Osteoclasts were transfected with B-DNA (B; 0.5 µg/mL), 5' triphosphate double-stranded RNA (53P; 1 µg/mL), polyinosinic-polycytidylic acid (pIC; 0.5 µg/mL), or Y-DNA (Y; 1 µg/mL) complexed with L2K, or were treated with L2K alone or challenged with LPS (5 ng/mL).  At 8 hours post-transfection, expression of RIG-I and cGAS was assessed by immunoblot analysis normalized to β-actin levels and representative immunoblots are shown (A-B).
